# Analysis of the Referral Rates of Newborn Hearing Screening Test According to Childbirth Delivery Methods in Neonatal Care Units

**DOI:** 10.3390/jcm10132923

**Published:** 2021-06-29

**Authors:** Ganghyeon Seo, Hyo Geun Choi, Sookyung Jang, Sun Choi, Sa Ra Lee, Su-Kyoung Park

**Affiliations:** 1 Department of Otorhinolaryngology-Head and Neck Surgery, Kangnam Sacred Heart Hospital, Hallym University, College of Medicine, 1, Singil-ro, Yeongdeungpo-gu, Seoul 07441, Korea; steemedak@gmail.com (G.S.); pandapanda@hallym.or.kr (S.J.); chl424@naver.com (S.C.); 2 Department of Otorhinolaryngology-Head and Neck Surgery, Hallym Sacred Heart Hospital, Hallym University, College of Medicine, 22, Gwanpyeong-ro 170beon-gil, Dongan-gu, Anyang 14068, Korea; pupen@naver.com; 3 Department of Obstetrics and Gynecology, Asan Medical Center, University of Ulsan, College of Medicine, 88, Olympic-ro 43-gil, Songpa-gu, Seoul 05505, Korea

**Keywords:** newborn hearing screening, auditory brainstem response, otoacoustic emissions, cesarean section, referral rate

## Abstract

It is known that neonates born by cesarean delivery (CD) may have higher referral rates than those born by vaginal delivery (VD) for newborn hearing screening (NHS). False-positive NHS results can increase costs and parental anxiety. This study analyzed the differences in NHS referral rates according to delivery methods in Level I, II, and III neonatal care units. A retrospective chart review was done for 2322 infants (4644 ears) with delivery records who underwent NHS between 2004 and 2017. The first NHS was performed immediately before discharge when the infant was in good condition via the automated auditory brainstem response (AABR) or automated otoacoustic emissions (AOAE). There were 98 neonates (196 ears) who underwent both AABR and AOAE simultaneously as the first NHS, 30 of which failed. We used a total of 4810 ears in this analysis. Of all enrolled ears, 2075 ears were of neonates born by CD, and 2735 ears were of neonates born by VD. A total of 2460 ears were from patients in Level III neonatal intensive care units (NICU) and 2350 ears were from Level I and II neonatal care units. The overall referral rate was higher in infants born via CD (4.5%) than VD (3.2%). In Level I and II neonatal intensive care units, the referral rate was significantly higher in those born via CD (3.0%) than via VD (1.4%). Further, based on the screening method, AABR (75.8%) was more frequently used than AOAE (24.2%), thereby revealing AABR’s higher referral rate in CD (2.9%) than in VD (1.2%). The referral rate of infants who underwent the NHS within three days of birth was higher in the CD group (3.0%) than in the VD group (1.3%). There was no significant difference in the referral rate depending on the delivery method when infants were hospitalized for more than four days or hospitalized in the NICU. The referral rate according to the delivery methods was significantly higher when the NHS test was performed for healthy newborns in the Level I and II neonatal care units born by CD within 72 h using AABR. Therefore, we recommend that the hearing screening test for newborns delivered by cesarean section be performed after 72 h of age. The results of this study may reduce the false-positive NHS results, unnecessary further tests, and parental anxiety.

## 1. Introduction

Early detection and intervention for newborn and infant hearing loss are crucial for age-appropriate language, communication, and cognitive development [1,2,3]. Therefore, in most developed countries, universal newborn hearing screening is conducted. In Korea, newborn hearing screening (NHS) tests have been covered by the National Health Insurance since October 2018 [4]. In South Korea, a low birth rate associated with increasingly delayed marriages is an important social problem [5]. It has become an important social issue that all children should be supported to overcome their disabilities and grow up to be active members of society. Consequently, it is increasingly important for the government to support newborn health [6]. Therefore, national newborn health screening systems, such as for hearing or congenital metabolic disorders, have targeted the early detection and treatment of children with disabilities. This has become essential in Korea [2,4,6].

International and Korean NHS guidelines recommend that screening tests be performed from 34 weeks of gestational age and within one month of birth at the adjusted chronological age and 24–72 h after birth before discharge [1,2,7,8]. Newborns born by cesarean delivery (CD) require NHS tests at least 24 h after birth to ensure that the ear canal is free of debris and discharge, which can be checked via a physical examination with an otoscope or tympanometry to decrease the false-positive rate and referral rate of NHS [2,7,9]. A study that examined NHS results according to delivery methods in healthy newborns hospitalized up to three days after birth recommended that in infants born via CD and when performing the NHS test with otoacoustic emissions, the pass rate increases only after the test is performed at least 48 h after delivery [10].

CD is an important delivery method, especially in an emergency. Absolute maternal indications of CD include severe antepartum hemorrhage due to placenta previa or abruptio placentae, major cephalopelvic disproportion, transverse lie, and brow presentation [11]. Fetal indications are situations in which neonatal morbidity and mortality could be decreased by CD. CDs for relative indications, in which CD is safest for the mother and newborn, and elective CDs are continuously increasing due to advanced maternal age in South Korea [11]. According to the National Survey on Fertility and Family Health and Welfare in 2018, 57.7% of natural births and 42.3% of CD were performed in South Korea [12]. In women aged 40–45 years, the CD rate was 64.8%, which is very high compared to other age groups [12]. The high CD rate could increase the probability of false-positive NHS results compared to vaginal delivery (VD), which could lead to a high referral rate.

Parents may experience stress when NHS results are ‘referred’, and the false-positive NHS results incur economic burden as well as the cost of performing additional screening and confirmatory hearing tests. Existing guidelines and the literature recommend that NHS in infants born by CD be implemented at least 24–48 h after birth to allow external auditory canal debris to clear. Recently, due to medical advances and insurance issues, the maternal hospitalization period has been getting shorter. Earlier hearing screening tests may be attributed to shorter maternal hospital stays after delivery. As the rate of CD is increasing, it may not be possible to secure sufficient hospitalization time to reduce the NHS false-positive rate [12].

The hospitalization period for newborns varies according to the neonatal care unit and characteristics of the newborn and accompanying diseases. In many countries, hospital-based neonatal care units are divided into different levels. A level I unit is referred to as a well newborn nursery, which provides basic neonatal care such as evaluation and postnatal care of healthy newborns and neonatal resuscitation. Level II includes specialty neonatal care of preterm infants with low-birth weight, resuscitation, and stabilization of preterm and ill-infants before transferring them to a neonatal intensive care unit (NICU) facility. Level III is the NICU for the subspecialty neonatal intensive care of infants who require mechanical ventilation, have undergone major surgery, or have severe congenital heart anomalies that require cardiopulmonary bypass [13]. Since the duration of hospitalization varies according to the neonatal care unit, it is necessary to analyze the NHS results that can be affected by the timing of the test after birth and the delivery mode. To the best of our knowledge, there is no report regarding the impact of delivery method on the referral rate considering the characteristics of the neonatal care units after NHS.

This study aimed to investigate the impact of delivery method (vaginal or cesarean) on the NHS referral rate in Level I and II neonatal care units and the Level III, NICU. We tried to analyze the factors associated with and the timing for an increased NHS pass rate in newborns born by CD.

## 2. Materials and Methods

### 2.1. Participants and Screening Methods

Long-term retrospective data were collected for 2322 infants hospitalized in the Hallym University Kangnam Sacred Heart Hospital with delivery records who had undergone NHS tests between 2004 and 2017. The first NHS was performed immediately before discharge if the infant was healthy with the automated auditory brainstem response (AABR) (MB11 BERAphone, MAICO Diagnostics, Berlin, Germany or Audioscreener, GSI, Eden Prairie, MN, USA) or Automated Otoacoustic Emissions (AOAE) measurements (Audioscreener, GSI, Eden Prairie, MN, USA). Two NHS devices were randomly used regardless of the neonatal level. In Level I and II neonatal care units, either AOAE or AABR was performed, and in Level III, AABR alone or AABR and AOAE were performed simultaneously. Since 2011, in Level III NICU newborns, we tried to perform two NHS tests simultaneously due to the possibility of auditory neuropathy, but this was not performed in all NICU newborns.

AABR set a sound stimulus intensity of 35 dB nHL. A total of 98 neonates (196 ears) underwent both AABR and AOAE as the first NHS. For the total infants, 30 ears failed the NHS test due to their own problems, such as moving or crying. Therefore, a total of 4810 ears were included in this analysis. The results of the second and subsequent NHS results were excluded from this study.

### 2.2. Classification of Groups and Methods

Eligible ears were divided into a VD group (2075 ears) and CD group (2735 ears). The following variables were obtained from the medical records of each group: NHS result (pass or refer), sex, birth weight, gestational weeks at birth, hospitalization location (Level I and II neonatal care units or Level III, NICU), admission duration, NHS days after birth, and NHS method (AABR or AOAE).

All ears were classified into four subgroups according to gestational weeks and birth weight. The criterion gestational age of each subgroup was 35 weeks because AABR should be performed at least 34 weeks after birth based on the adjusted chronological age and the gestational age was ≥35 weeks per the criteria for discharge from the NICU by the Ministry of Health and Welfare [12]. The birth weight, which is the criterion for subgroup classification, was based on 2.9 kg, the 25th percentile of the 2017 Korean national growth charts for NICU infants [14].

### 2.3. Statistical Analysis

Quantitative variables such as birth weight and admission days were described by the number of non-missing values, mean, and standard deviation. Qualitative variables such as sex and NHS method were described with the number and percentage of ears. Missing values were not included in the calculation. All statistical analyses were performed using SPSS (version 25.0, IBM Corp., Armonk, NY, USA). Group comparison for qualitative data was performed by the Pearson chi-square test. For quantitative data, we used Student’s *t*-test. *p*-values < 0.05 were considered statistically significant.

## 3. Results

### 3.1. Characteristics of NHS Ears by Delivery Method

A total of 4810 ears were enrolled in this study. A total of 2075 ears (43.1%) were in the CD group, and 2735 ears (56.9%) in the VD group (Table 1). Based on infants’ sex, 1172 ears (586 infants, 56.5%) of male newborns were included in the VD group and 1494 ears (747 infants, 54.6%) were included in the CD group (*p* > 0.05). The mean body weight at birth was 2.53 ± 0.86 kg. Birth weight was significantly lower in the CD group at 2.67 ± 0.81 kg than in the VD group, at 2.42 ± 0.88 kg (*p* < 0.0001). 

A total of 1230 infants (2460 ears, 51.1%) were admitted at the Level III, NICU, and a total of 1175 infants (2350 ears, 49.9%) were admitted at Level I and II neonatal care units, respectively. The proportion of CD in the Level III, NICU was significantly higher than that of Level I and II neonatal care units (*p* < 0.0001) (Table 1). For all infants, the overall mean hospital stay was 20.39 ± 31.24 days. The mean hospital stay was longer in the CD group (22.27 ± 30.60 days) than in the VD group (17.92 ± 31.91 days) (*p* < 0.05) (Table 1).

### 3.2. Referral Rates According to Gestational Weeks and Birth Weight

**Figure 1:**
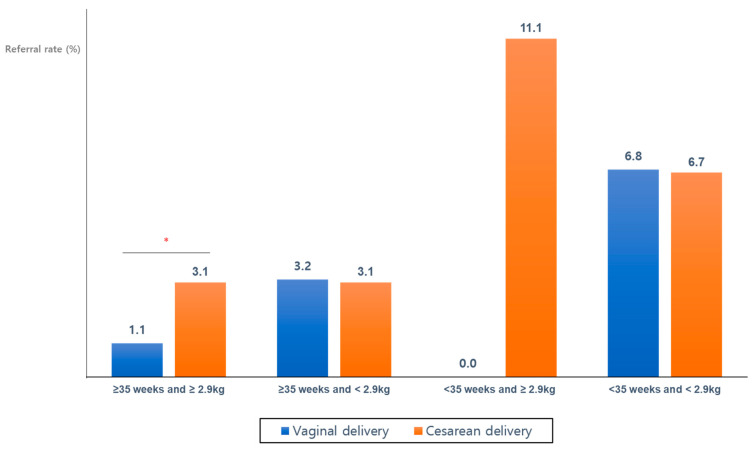
Referral rates of newborn hearing screening tests according to gestational weeks, birth weight, and delivery method. The referral rate was statistically significantly higher in those born via CD, when the infants’ gestational age was ≥35 weeks and when the birth weight was ≥2.9. * *p* < 0.05 in the Pearson chi-square test and Fisher’s exact test.

**Figure 2:**
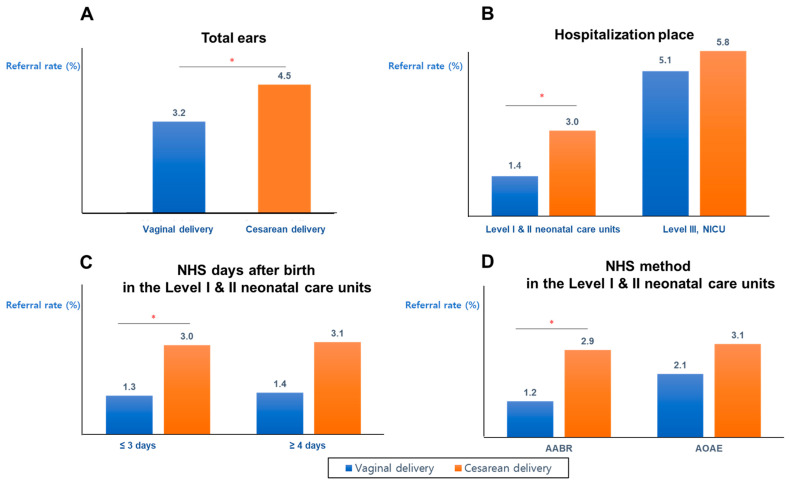
Referral rates of newborn hearing screening test of total ears (**A**), hospitalization place (**B**), NHS days after birth in Level I and II neonatal care units (**C**), and NHS method in the Level I and II neonatal care units (**D**) according to the delivery methods. For the total enrolled ears, the referral rate of those born via CD was statistically significantly higher than that of those born via VD (**A**). The NHS referral rate was significantly higher in CD than in VD in Level I and II neonatal care units (**B**). When the screening was performed within ≤3 days after birth, the referral rate of the CD group was significantly higher than that of VD group (**C**). In the Level I and II neonatal care units, the referral rate of AABR was significantly higher than that of AOAE. * *p* < 0.05 in the Pearson chi-square test and Fisher’s exact test. AABR, automated auditory brainstem response; AOAE, automated otoacoustic emission; CD, cesarean delivery; NHS, newborn hearing screening; NICU, neonatal intensive care unit; VD, vaginal delivery.

For the analysis of referral rate according to the subgroup stratified by both gestational weeks (≥35 weeks or <35 weeks) and birth weight (≥2.9 kg or <2.9 kg), only the group of ‘≥35 weeks gestation and ≥2.9 kg birth weight’ had a significant difference in referral rate between the two delivery methods; the referral rate was significantly higher in the CD group (3.1%) than in the VD group (1.1%) (*p* = 0.003). On the other hand, the referral rates were not significantly different between two delivery methods in the other subgroups, such as ‘gestational weeks ≥35 and birth weight <2.9 kg’, ‘gestational weeks <35 and birth weight ≥2.9 kg’, and ‘gestational weeks <35 and birth weight <2.9 kg’ (*p* > 0.05) (Table 2 and Figure 1). For all enrolled ears, the referral rate was higher in the CD group (4.5%) than in the VD group (3.2%) (*p* = 0.022) (Table 2, Figure 2).

### 3.3. Referral Rates Stratified by NHS Methods

For all enrolled ears that used an AABR device for the hearing screening test, the referral rates in the CD and VD groups were 3.7% and 2.7%, respectively (*p* = 0.095). There were no differences in the referral rates in the AOAE cases (6.9% and 5.1% for CD and VD, respectively, *p* = 0.205) (Table 2). However, in the case of infants admitted in Level I and II neonatal care units, the NHS referral rate tested by the AABR was significantly higher in the CD group (2.9%) than in the VD group (1.2%) (*p* = 0.010) (Table 2, Figure 2).

### 3.4. Referral Rates of Infants Admitted into Level I and II Neonatal Care Units and Level III, NICU

In Level I and II neonatal care units, the referral rate was higher in the CD group (3.0%) than in the VD group (1.4%) (*p* = 0.007). However, the referral rates were not different in infants admitted into the NICU (5.8% in the CD group and 5.1% in the VD group (*p* > 0.05) (Table 2, Figure 2B).

### 3.5. Referral Rates Stratified by NHS Timing after Birth in Level I and II Neonatal Care Units

The referral rate was higher in the CD group (3.0%) in infants admitted in Level I and II neonatal care units who underwent the NHS within three days after birth than in the VD group (1.3%) (*p* = 0.035). However, the referral rate appeared to be higher in the CD group (3.1%), although it was not significantly different in the VD group (1.4%) among infants admitted in Level I and II neonatal care units who underwent the NHS more than four days after birth (Table 2, Figure 2).

## 4. Discussion

This study analyzed the impact of delivery method (vaginal or cesarean) on the NHS referral rates among newborns born and admitted in Level I and II neonatal care units and Level III, NICU at a single university-based hospital. In this study, the rate of using AABR as an NHS test (75.8%) was higher than that of using AOAE (24.2%) for all enrolled infants. The reason for this is that the current international and Korean guidelines recommend the use of the AABR test, which has high specificity and sensitivity, for all neonates including newborns in the NICU, to avoid missing cases of auditory neuropathy [1,2,8,15]. Most reports analyzed the impact of the delivery method on the high referral rate associated with the NHS false-positive rate using the AOAE method among term babies or infants in a Level I neonatal care unit, well-baby nursery [10,16]. However, our study analyzed both AOAE and AABR among Level I and II neonatal care units and Level III, NICU.

The CD rate has been increasing worldwide [12,17,18]. The CD rate in South Korea is the highest in the world, except for Turkey, according to the 2019 Organization for Economic Co-operation and Development report [19]. Following a sharp rise from 4% in 1980 to 40% in 2000 [17]. The overall CD rates in South Korea were 45.0% in 2017 and 47.3% in 2018 according to the National Health Screening Statistical Yearbooks for 2017 and 2018 [12,17,20]. That is, approximately half of Korean newborn babies were delivered by caesarean section. If the NHS result of a newborn born by CD is ‘refer’, which might be a false-positive result, it may have occurred if the screening test was conducted too early after birth (within 48 to 72 h). Therefore, the drum and ear canal should be examined in such cases.

Neonates born by CD have higher NICU admission rates than those born by VD due to a high rate of respiratory morbidity, including respiratory distress syndrome or mild transient tachypnea, regardless of gestational age [11]. Our results also showed a higher NICU admission rate in the CD group than in the VD group. VD offers many respiratory, hematologic, and immunologic benefits, as the baby is delivered naturally and can physiologically adapt to the external environment. VD increases external pressure on the ear during the second stage of labor. During this period between full cervical dilatation and birth, the baby’s head is partially squeezed when passing through the cervical and vaginal canals [21]. The duration of the second stage varies and is affected by parity. The median duration of this stage is 14–66 min (0.2–1.1 h), with 95th percentile thresholds of 65–138 min (1.1–2.3 h), 6–12 min (0.1–0.2 h) in nulliparous women, and 58–76 min (1.0–1.3 h) in parous women [20]. This stage can squeeze the amniotic fluid from the baby’s ear [21,22]. VD can positively affect ear function with lower NHS referral rates compared with CD.

Many guidelines recommend the NHS test be performed at least 24 h after birth to decrease “false-positive” rates [1,2,7,9]. In a study evaluating the NHS test performance of wideband acoustic transfer functions in a universal NHS program, changes in sound conduction were observed during the first two days after birth due to middle ear effusion or ear canal factors, such as debris after birth [23]. In a study of the NHS results of 2784 infants in public and private hospitals in India, the false-positive rate in the first NHS in those delivered via VD was significantly higher than in those delivered via CD, and the referral rate significantly decreased with screening age. However, these results cannot be universally applied to other country hospitals because poor NHS quality control and the delivery state of public hospitals in this study may have affected the results. In a study which analyzed the NHS test results of 1653 healthy newborns with birth weights of 2501–4000 g, born after 35 weeks, and who underwent NHS by the AOAE test within 48 h after birth, the referral rate was three times higher in babies born by CD than in those born by VD [24]. A Turkish study reported no difference in the referral rate according to the delivery mode among 2653 healthy newborns who underwent NHS using the transient evoked otoacoustic emissions test [25]. The false-positive rate was 81.9% in the tests performed within the first few days after birth and 14.5% in the tests performed 15 days after birth. The authors suggest a positive result on the first NHS should be repeated [25]. However, this study included only 212 newborns who underwent their first NHS who were delivered by CD. The limitation of the Turkish NHS study was that the number of CD cases was too small [25].

Our study performed a subgroup analysis on newborns with over or below 35 weeks gestational age, considering the guidelines’ recommendations that newborns should be performed NHS with AABR test at the age of more than 34 gestational weeks. We also performed subgroup analysis on babies based on the 2.9 kg birth weights, which corresponds to the 25th percentile of the 2017 Korean National Growth Charts for children’s data [14]. The results of the subgroup analysis showed that the referral rate was significantly higher in the CD group (3.1%) than in the VD group (1.1%) in the infants of the subgroup, ‘≥35 weeks and ≥2.9 kg’. In a subgroup analysis, there was no newborn in VD group of ‘a gestational age < 35 weeks and birth weight ≥ 2.9 kg’ and the referral rate increased to 11.1% in the CD group (2/18 ears, one infant), which result involved a special case of large babies and maternal gestational diabetes, as stated in the literature review [26].

In a study comparing the referral rate between the first AOAE tested within 48 h (*n* = 560) or after 48 h (*n* = 566), this rate was significantly higher in the group tested within 48 h (20.5%) than in the group tested 48 h after birth (3.4%) [16]. In our study, the referral rate was not different according to the delivery method in babies tested by AOAE. The referral rate was significantly higher in babies delivered by CD and tested by the AABR in the Level I and II neonatal care units. This may be due to the relatively small number of infants (24.2%) tested by the AOAE. Further study including more babies tested by the AOAE is warranted.

The rate of CD was higher in the NICU in our study, although the referral rates did not differ between babies born by CD or VD in babies admitted to the NICU. This may be because NICU babies typically stay in the hospital for more than three days, with mean hospital stays of 35.5 days in our study. Relatively long hospital stays allow the amniotic fluid or debris such as vernix caseosa to flow out from the ear canal. Transient otitis media can also be resolved during this time. These factors could be why there are no significant differences by the delivery method in reducing the high referral rate associated with CD. The high referral rate in babies born by CD in the Level I and II neonatal care units was noted in those who underwent the test within three days after birth in this study; however, the difference was not noted in babies in the Level I and II neonatal care units who underwent the test later than the fourth day after birth. Based on this result, we suggest that the optimal timing of NHS for babies in the WBN delivered by CD is at least 72 h after birth, a criterion longer than the previously recommended 24 or 48 h after birth.

This study analyzed newborn babies delivered in a university-based hospital over 14 consecutive years. The limitations of our study were as follows. First, the number of babies who underwent NHS by the AOAE test was too small to evaluate the previously reported high referral rates associated with the AOAE test. Second, there were only some data regarding the second NHS results and formal confirmatory testing with diagnostic auditory brainstem response threshold test. We therefore could not calculate the false-positive rate. Further large-scale, multi-center studies will be needed to complement these limitations. However, this study can be meaningful to analyze the impact of the delivery methods on the referral rates of NHS, not only for the Level I and II neonatal care units, but also NICU, and to present the appropriate timing and circumstances to reduce the false-positive ‘refer’ results of the infants born through CD.

## 5. Conclusions

In conclusion, the referral rate according to the delivery method was significantly higher when the NHS test was performed for newborns in Level I and II neonatal care units with CD within 3 days after birth using AABR. Therefore, we recommend that the hearing screening test for newborns delivered by cesarean section be performed after 72 h of age. The results of this study may help reduce the NHS false-positive, unnecessary testing, and parental anxiety.

## Figures and Tables

**Table 1:** Characteristics of newborn hearing screening tests classified by delivery method.

Variables	Total (4810 Ears)	Vaginal Delivery (2075 Ears)	Cesarean Delivery (2735 Ears)	*p*-Value (Both Delivery Methods)
Male sex, ears (% of male)	2666 (55.4)	1172 (56.5)	1494 (54.6)	0.199
Birth weight (kg)	2.53 ± 0.86	2.67 ± 0.81	2.42 ± 0.88	<0.0001 *
Gestational weeks at birth	35.54 ± 8.29	36.11 ± 4.01	35.11 ± 10.39	0.311
Level I, II neonatal care unit, ears (%)	2350 (49.9)	1110 (53.5)	1240 (45.3)	<0.0001 *
Level III, NICU admission, ears (%)	2460 (51.1)	965 (46.5)	1495 (54.7)	
Admission days	20.39 ± 31.24	17.92 ± 31.91	22.27 ± 30.60	0.003 *
NHS days after birth	17.5 ± 28.02	15.88 ± 27.97	18.73 ± 28.00	<0.0001 *
NHS method				
AABR, ears (%)	3644 (75.8)	1620 (78.1)	2024 (74.0)	0.001 *
AOAE, ears (%)	1164 (24.2)	454 (21.9)	710 (26.0)	

A total of 2322 infants participated, and 98 neonates (196 ears) underwent both AABR and AOAE simultaneously as the first NHS. For the total infants, 30 ears failed the NHS test due to their own problems. Therefore, a total of 4810 ears were included in this analysis. * *p* < 0.05 in the Pearson chi-square test and Fisher’s exact test for categorical variables, or independent *t*-tests for continuous variables. AABR, automated auditory brainstem response; AOAE, automated otoacoustic emission; NHS, newborn hearing screening; NICU, neonatal intensive care unit; SD, standard deviation.

**Table 2:** Referral rates of newborn hearing screening tests classified by delivery method.

Variables	Number of Ears: Referred Ears/Total Ears (%, Referral Rate)		*p*-Value
	Vaginal Delivery	Cesarean Delivery	
Total ears	67/2075 (3.2)	124/2735 (4.5)	0.022 *
By gestational weeks and birth weight			
≥35 weeks and ≥2.9 kg	11/976 (1.1)	26/846 (3.1)	0.003 *
≥35 weeks and <2.9 kg	16/496 (3.2)	27/870 (3.1)	0.901
<35 weeks and ≥2.9 kg	0/12 (0.0)	2/18 (11.1)	0.232
<35 weeks and <2.9 kg	39/574 (6.8)	67/996 (6.7)	0.959
By NHS method			
AABR	44/1620 (2.7)	75/2024 (3.7)	0.095
AOAE	23/454 (5.1)	49/710 (6.9)	0.205
By hospitalization place			
Level I and II neonatal care units	15/1110 (1.4)	37/1240 (3.0)	0.007 *
By NHS days after birth			
<4 days	8/624 (1.3)	21/706 (3.0)	0.035 *
≥4 days	7/486 (1.4)	16/534 (3.1)	0.095
By NHS method			
AABR	11/922 (1.2)	25/854 (2.9)	0.010 *
AOAE	4/188 (2.1)	12/386 (3.1)	0.598
Level III, NICU	52/964 (5.4)	87/1494 (5.8)	0.653

Missing values were excluded. * *p* < 0.05 in the Pearson chi-square test and Fisher’s exact test for categorical variables, or independent *t*-tests for continuous variables. AABR, automated auditory brainstem response; AOAE, automated otoacoustic emission; NHS, newborn hearing screening; NICU, neonatal intensive care unit.

## Data Availability

All datasets analyzed in this study are available from the co-corresponding author S-K Park (ashock@daum.net) on reasonable request.
